# Aristolochic Acid I Induced Autophagy Extenuates Cell Apoptosis via ERK 1/2 Pathway in Renal Tubular Epithelial Cells

**DOI:** 10.1371/journal.pone.0030312

**Published:** 2012-01-20

**Authors:** Youjia Zeng, Xiao Yang, Juan Wang, Jinjin Fan, Qingyu Kong, Xueqing Yu

**Affiliations:** Department of Nephrology, The First Affiliated Hospital, Sun Yat-sen University, Key Laboratory of Nephrology, Ministry of Health, Guangzhou, Guangdong, China; University of Houston, United States of America

## Abstract

Autophagy is a lysosomal degradation pathway that is essential for cell survival and tissue homeostasis. However, limited information is available about autophagy in aristolochic acid (AA) nephropathy. In this study, we investigated the role of autophagy and related signaling pathway during progression of AAI-induced injury to renal tubular epithelial cells (NRK52E cells). The results showed that autophagy in NRK52E cells was detected as early as 3–6 hrs after low dose of AAI (10 µM) exposure as indicated by an up-regulated expression of LC3-II and Beclin 1 proteins. The appearance of AAI-induced punctated staining of autophagosome-associated LC3-II upon GFP-LC3 transfection in NRK52E cells provided further evidence for autophagy. However, cell apoptosis was not detected until 12 hrs after AAI treatment. Blockade of autophagy with Wortmannin or 3-Methyladenine (two inhibitors of phosphoinositede 3-kinases) or small-interfering RNA knockdown of Beclin 1 or Atg7 sensitized the tubular cells to apoptosis. Treatment of NRK52E cells with AAI caused a time-dependent increase in extracellular signal-regulated kinase 1 and 2 (ERK1/2) activity, but not c-Jun N-terminal kinase (JNK) and p38. Pharmacological inhibition of ERK1/2 phosphorylation with U0126 resulted in a decreased AAI-induced autophagy that was accompanied by an increased apoptosis. Taken together, our study demonstrated for the first time that autophagy occurred earlier than apoptosis during AAI-induced tubular epithelial cell injury. Autophagy induced by AAI via ERK1/2 pathway might attenuate apoptosis, which may provide a protective mechanism for cell survival under AAI-induced pathological condition.

## Introduction

Autophagy is a cellular process of “self-digestion” through lysosomal degradation pathway [1], [2], which is a major regulated mechanism for degrading long-lived proteins and organelles. Early studies confirmed that autophagy was an adaptive responder of cells to nutrient deprivation, i.e. to ensure minimal housekeeping functions. In the last decade, a tremendous gain of knowledge on autophagy has been achieved, showing that cell injury or accumulation of damaged organelles/membranes, intracellular inclusions may activate the autophagic pathway [3]. It was demonstrated cisplatin [4] or cyclosporine A [5] could induce autophagy in renal tubular epithelial cells, which occurred prior to apoptosis, and played a protective role during kidney injury. However, inappropriate activation of autophagy may facilitate cell death. Previous studies considered type I programmed cell death (apoptosis), type II (autophagy) and type III (necrosis) represented the major types of programmed cell death that served to trigger cell death [6]. The visualization of autophagosomes in dying cells led to the belief that autophagy is a nonapoptotic form of programmed cell death. In terms of autophagic death of renal tissues, it was found that autophagy is another potential mechanism of nephron loss in obstructive uropathy [7]. These emerging studies suggested autophagy occurrence was widespread and important, but its effect on cell fate was controversial.

Aristolochic acids (AAs) are a family of structurally related nitrophenanthrene carboxylic acids contained in the plant extract of the aristolochia species such as *A. fangchi*, *A. clematits*, and *A. manshuriensis* [8]. The major components of AAs are AAI and AAII [9]. AAs were shown to be toxic to renal tubular epithelium cells [3], [10], [11]. In terms of pathophysiology of aristolochic acid nephropathy (AAN), apoptosis caused by AAs has been confirmed that leads to subsequent irreversible proximal tubular atrophy [12], [13]. Although autophagy and apoptosis are coordinated processes for cell fate, the role of autophagy in AAs-induced renal tubular epithelial cell (RTEC) injury remains unclear. Based on these findings, we first raised the hypothesis that autophagy may occur and play an important role in AA-induced renal proximal tubular cell injury.

The complex molecular mechanism of autophagy is responsible for its complicated and multiple pathways under various conditions. Of note, these signaling pathways may cross talk and regulate at different levels in the autophagic cascade [14]. It was reported that mitogen-activated protein kinases (MAPKs), including ERK1/2, p38, and JNK, involved in autophagy induced by nutrient starvation [15], [16]. AAI-induced oxidative DNA damage associated with ERK1/2 and p38 activation in human promyelocytic leukemia cells (HL-60) [17]. However, the signaling mechanisms regulating AAs-induced autophagy are not defined. In this study, we investigated the role of autophagy and related signaling pathway during progression of AAI-induced injury to renal tubular epithelial cells (NRK52E cells). We provided evidence that autophagy occurred earlier than apoptosis during AAI-induced tubular epithelial cell injury. Autophagy induced by AAI via ERK1/2 pathway could attenuate apoptosis, which may provide a protective mechanism for cell survival under AAI-induced pathological condition.

## Materials and Methods

### Antibodies and reagents

Dulbecco's modified Eagle's medium/F12 (DMEM/F12) and fetal bovine serum (FBS) were purchased from Gibco BRL (GIBCO-BRL, Grand Island, NY). AAI was purchased from National Institute for the Control of Pharmaceutical and Biological Products (Beijing, China) and was prepared as a stock solution of 8.3 mg/ml in 100% dimethyl sulfoxide (DMSO) for cell culture. Anti-LC3 was purchased from Novus Biologicals, LLC (Novus Biologicals, Littleton, CO, USA) and anti-Beclin 1, anti-Atg7 from Abcam.Ltd (Abcam, Cambridge, UK). Anti-cleaved caspase3, anti-β-Actin, anti-phospho-P44/42 MAP Kinase, anti-P44/42 MAP Kinase, anti-SAPK/JNK, anti-phospho-SAPK/JNK, anti-p38MAP Kinase and anti-phospho-p38 MAP Kinase were from Cell Signaling Technology.Inc (CST, Danvers, Mass, USA). All secondary antibodies from Santa Cruz Biotechnology,® inc. (Santa Cruz, California, USA) Lipofectamine 2000 was from Invitrogen Life Techonologies (Invitrogen, Carlsbad, CA). Wortmannin, E64d, Pepstatin A, 3-Methyladenine (3-MA), Annexin V-FTIC Apoptosis Detection Kit and U0126 were from Sigma-Aldrich Co. (Sigma, St Louis, MO, USA).

### Cell culture and AAI treatment

Normal rat renal tubular epithelial cells (TECs) line, NRK52E, was originally obtained from ATCC, USA and maintained in DMEM/F12 containing 10% FBS at 37°C with 5% CO_2_. The cells were seeded in 35-mm dishes at a density of 1×10^6^ cells per dish and subconfluent cells were used for the experiments. The cells were incubated either without or with AAI at various concentrations for different time period as indicated as follow. In preliminary studies, the suitable concentration and optimum exposure time of AAI were determined as 10 µM for 0 to 24 hrs. To observe the effects of inhibitors, cells were treated with the inhibitor Wortmannin (20 nM) [18], E64d(10 µg/ml)+Pepstatin A (10 µg/ml), 3 MA(5 mM) or U0126 (5 µM) [19] for 15 min, respectively, before addition of AAI according to the results of our preliminary tests.

### Transient transfection

The GFP-LC3 fusion plasmid was kindly provided by Professor Zhu Xiaofeng. NRK52E cells were plated at a density of 2×10^5^ on a coverslip and cultured up to 60% confluence. Transfection was carried out with Lipofectamine 2000 as manufacturer's recommendation and 2 µg/ml GFP-LC3 plasmid DNA in each dish was used. After incubation in Opti-MEM medium for 5–6 hrs, the cells were incubated in DMEM/F12 containing 10% FBS medium again. When cells reached 90% confluence, AAI was added into culture medium. The transfection efficiency for NRK52E cells was 20%, approximately.

### RNA interference

As we known, Beclin 1 and its binding partner class III phosphoinositide 3-kinase (PI3K), also named Vps34, are required for the initiation of the formation of autophagosome during autophagy. The following sequence was used for short interfering RNA (siRNA) against Beclin 1: sense strand 5′-GAUUGAAGACACAGGAGGC-3 [20]. For siRNA against Atg7, the following sequence was used: 5′-GCAUCAUCUUUGAAGUGAA-3′ (Sigma). For a control siRNA, the following sequences were used: sense strand 5′-UUC UCC GAA CGU GUC ACG UTT-3′, anti-sense 5′-ACG UGA CAC GUU CGG AGA ATT-3′. siRNA was transiently transfected using Lipofectamine 2000.

### Cell lysis, SDS-PAGE and western blotting

Cells were lysed on ice for 15 min in 1×RIPA lysis buffer. Protein concentration was measured using the Bradford assay. The amount of protein was approximately 30∼60 µg (concentration measured by Bradford method: 1.5∼3 mg/ml) per sample. Equal amounts of cell extracts were boiled for 5 min in the presence of 1% 2-mercaptoethanol and 2% sodium dodecyl sulfate (SDS). Samples were electrophoresed on 15% SDS-poly-acrylamide gels, and then transferred to polyvinylidene difluoride (PVDF) membrane. Membranes were blocked with 5% (wt/vol) milk proteins/Tris-buffered saline and incubated overnight at 4°C with the primary antibody: rabbit anti-LC3 (2 µg/ml), rabbit anti-Beclin 1 (1 µg/ml), rabbit anti-Atg7 (1 µg/ml), rabbit anti-cleaved caspase 3 (2 µg/ml), anti-phospho-P44/42 MAP Kinase (2 µg/ml), anti-P44/42 MAP Kinase (2 µg/ml), anti-SAPK/JNK (2 µg/ml), anti-phospho-SAPK/JNK (2 µg/ml), anti-p38MAP Kinase (2 µg/ml) and anti-phospho-p38 MAP Kinase (2 µg/ml). Bound antibodies (dilution:1∶2000) were detected with appropriate secondary antibodies and enhanced chemiluminescence. Same blots were probed with anti-β-Actin to monitor protein loading and transferring. A densitometric quantification of western blot signal intensity of membranes was performed.

### Autophagy assays

Autophagy was examined by analyzing the formation of fluorescent puncta of autophagosomes in GFP-LC3 transfected cells and immunoblot analysis of LC3-II and Beclin 1, another essential autophagy related protein. In fluorescence microscopy experiments, NRK52E cells were transiently transfected with GFP-LC3 plasmid and treated with AAI as described above. After treatment, the cells were fixed with 4% paraformaldehyde. Autophagy was evaluated by examining the punctate forms (type II) of the autophagy marker LC3 based on GFP-LC3. Quantitation of autophagy was done based on the percentage of GFP-LC3-positive autophagic vacuoles or cells with LC3 punctate dots. Twenty fields of ×600 magnification with 20 to 30 GFP-labeled green cells per field were counted in each condition [21].

### Cell apoptosis determination

All cells under various experimental conditions were harvested with PBS containing 0.05 trypsin and rinsed with PBS. Apoptosis cells were quantitated by staining with Annexin V-FITC and Propidium iodide (PI), and then measured with flow cytometry (FCM) according to the protocol of the AnnexinV-FITC Apoptosis Detection Kit. For morphology of apoptotic cells, treated cells on the coverslips were fixed with 4% paraformaldehyde (PFA) and stained with 2 µg/ml Hoechst 33342. Nuclear morphology was examined by fluorescence microscopy. Immunoblot assay was used for cleaved-caspase3 analysis.

### Statistics

Qualitative data was representative of at least three experiments and was expressed as means ±SEM. Statistical analysis was conducted using the GraphPad Prism software. Statistical differences in multiple groups were determined by multiple comparisons with analysis of variance followed by Tukey's post-tests. Statistical differences between two groups were determined by two-tailed unpaired Student's *t*-test. *P<0.05* was considered significant difference.

## Results

### Autophagy was induced in NRK52E cells in response to AAI treatment

**Figure 1:**
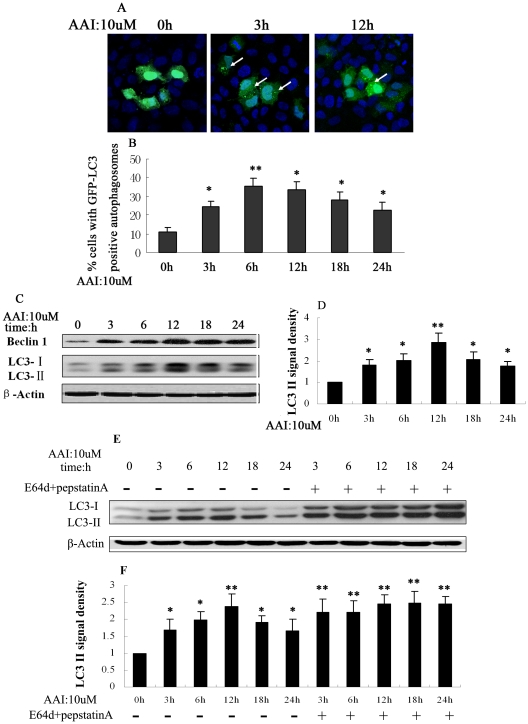
Autophagy induced by AAI (10 µM) in NRK-52E cells. NRK-52E cells were transiently transfected with GFP-LC3 plasmid. After 0 to 24 hrs of AAI (10 µM) incubation, the cells were fixed and analyzed the formation and distribution of GFP-LC3 puncta by immunofluorescence. **A**, Representative images (×600). Arrows indicated GFP-LC3 puncta (green). Nuclei (blue) were stained by Hoechst33342. **B**, Percentage of cells with GFP-LC3 puncta. **C**, Western blot showed an increase in Beclin 1 and LC3-II at early stage of AAI incubation. **D**, Densitometric analysis of LC3-II in Fig. 1C. The protein expression level of control (0 hr) group was arbitrarily set as 1 in each blot, and the signals of other conditions in the same blot were normalized with the control to indicate their protein expression level. **E**, Western blot showed an continue increase in LC3-II with the lysosomal inhibitors E64d (10 µg/ml)+Pepstatin A (10 µg/ml) after AAI incubation. **F**, Densitometric analysis of LC3-II in Fig. 1E. **B, D** and **F** were expressed as means±SEM of three independent experiments. *and**denote *p<0.05* and *p<0.001*, respectively, when compared to control condition.

A low concentration of AAI (10 µM) was selected according to the results of MTT assay (data not shown) and the autophagy occurrence after AAI exposure in NRK52E cells was observed by detecting expression of GFP-LC3. Following AAI exposure, the transfected cells showed distributed puncta at 3 hrs which last later periods, while control transfected cells without AAI treatment showed a diffuse distribution of green fluorescence (Fig. 1A). Cell counting showed that 6 to 12 hrs of AAI exposure increased GFP-LC3 punctuate cells from the basal level of 10% to 35% (Fig. 1B). LC3-II formation was also tested by western blot analysis. AAI incubation induced a time-dependent accumulation of Beclin 1 and LC3-II in NRK52E cells, which appeared at 3 hrs and increased dramatically at 12 hrs, then decreased slightly thereafter (Fig. 1C and 1D). E64d and pepstatin A could inhibit degradation of LC3-II partially. The Fig. 1E and 1F showed the lysosomal inhibitors significantly increased LC3-II accumulation of NRK52E cells during incubation of AAI at each time point. These findings indicated that AAI didn't block autophagic flux, but induced the autophagic activity.

### Apoptosis followed tubular cell autophagy in response to AAI treatment

**Figure 2:**
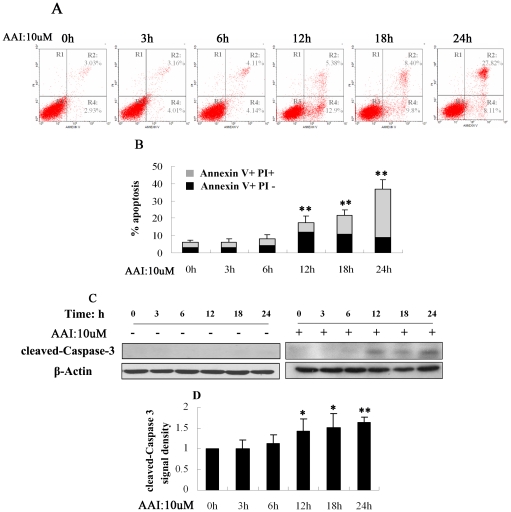
Apoptosis induced by AAI (10 µM) in NRK-52E cells. **A**, Representative annexinV/PI flow cytometry analysis at different AAI incubation time. The percentage of early apoptotic cells was identified with Annexin V+PI− (R4) and late apoptotic and necrotic cells was identified with AnnexinV+PI+ (R2). **B**, Quantitative analysis of AnnexinV+PI− and AnnexinV+PI+ NRK52E cells by flow cytometry. **C**, Western blot showed an increase in cleaved Caspase-3 after AAI incubation from 12 hrs to 24 hrs, while cleaved Caspase-3 didn't increase (0∼24 hrs) without AAI incubation. **D**, Densitometric analysis of cleaved Caspase-3 in Fig. 2C. **B** and **D:** Data was expressed as means±SEM of three independent experiments. *and**denote *p<0.05* and *p<0.001*, respectively, when compared to control condition.

Cell apoptosis at the same condition described above was measured by FCM. As shown in Fig. 2A, during early stage of AAI treatment (3–6 hrs), most cells were normal and dynamic. Apoptotic cells were not induced until 12 hrs and increased thereafter in a time-dependent way (Fig. 2A and 2B). At 12 h, approximate 15% cells were early apoptotic cells (Fig. 2A, Region 4), while small amount (5.38±1.29%) of late apoptotic cells (Fig. 2A, Region 2) was detected. After 12 hrs of AAI treatment, early apoptotic and normal cells were dying gradually. Over 25% late apoptotic and necrotic cells occurred at 24 hrs. Immunoblot analysis results proved that AAI did not induce significant up-regulated expression of cleaved-caspase 3 (17 kDa) in NRK52E cells until 12 hrs of incubation (Fig. 2C).

### Inhibition of AAI-induced autophagy with Wortmannin increased apoptosis in NRK52E Cells

**Figure 3:**
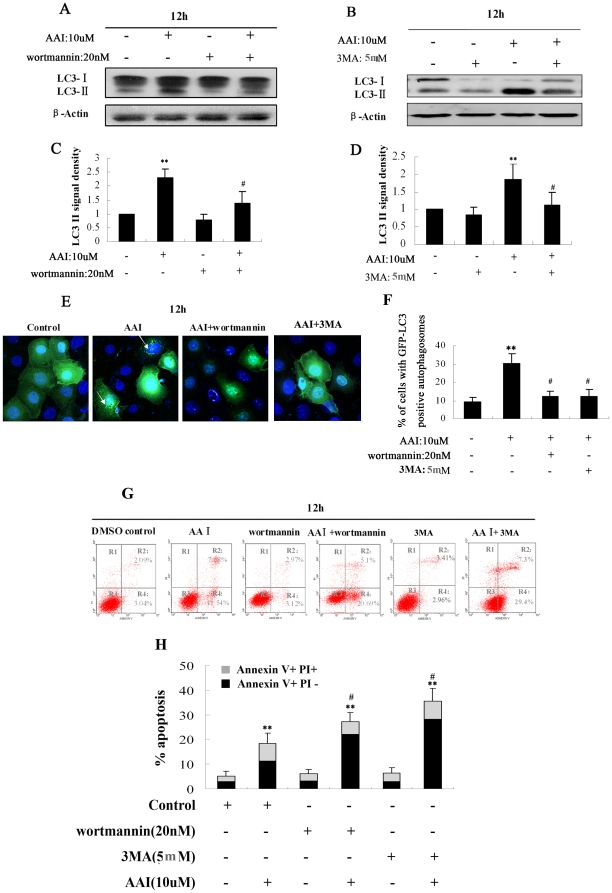
Inhibition of autophagy by Wortmannin and 3-MA increased apoptosis during AAI incubation of NRK52E cells. NRK52E cells were incubated for 12 hrs in the absence (DMSO control), or presence of AAI (10 µM), Wortmannin (20 nM), 3 MA (5 mM) or AAI plus Wortmannin, AAI plus 3 MA. Wortmannin and 3-MA blockaded the autophagy and increased apoptosis induced by AAI. **A** and **B:** Western analysis using antibodies against LC3-II or *β*-Action. **C** and **D:** Densitometric analysis of LC3II in Fig. 3A and 3B. NRK52E cells were transiently transfected with GFP-LC3 plasmid, then were subjected to the same conditions as described. **E**, Representative images (×800). Arrows indicated GFP-LC3 puncta (green). Nuclei (blue) were stained by Hoechst33342. **F**, Graphs represent quantitation analysis of the percentage of cells with GFP-LC3 positive autophagosomes. **G**, Representative annexinV/PI flow cytometry analysis of NRK52E cells. **H**, Graphs represented quantitation of analysis of AnnexinV+PI− and AnnexinV+PI+ NRK52E cells by flow cytometry. **C**, **D**, **F** and **H:** Data was expressed as means±SEM of three independent experiments. *and** denote *p<0.05* and *p<0.001*, respectively, when compared to control condition. ^#^denote *p<0.05*,compared to AAI group.

To investigate the effect of autophagy on cell apoptosis, Wortmannin and 3-Methyladenine (3-MA), two types of potent pharmacological inhibitors on autophagy, were used to suppress the autophagy induced by AAI. Our preliminary tests demonstrated that 15 min pretreatment with 20 nM Wortmannin or 5 mM 3-MA could block autophagy in NRK52E cells without significant cytotoxicity. As shown in figure 3A and 3B, Wortmannin or 3-MA pretreatment reduced the accumulation of LC3-II (Fig. 3A–D) and formation of GFP-LC3 puncta (Fig. 3E and 3F, as arrow indication). The effects of Wortmannin or 3-MA on apoptosis after AAI treatment were shown in figure 3G and 3H. Wortmannin or 3-MA itself scarcely induced cell apoptosis and cell death, but significantly increased apoptosis at 12 hrs after AAI exposure (AAI group: 18.8%; AAI plus Wortmannin group: 28.1%; AAI plus 3-MA:37.2%). These results suggested that suppression of autophagy by Wortmannin or 3-MA could increase AAI-induced injury in renal tubular epithelium cells.

### Short interfering RNAs (siRNA) against Beclin 1 accelerated AAI-induced apoptotic death in NRK52E cells

**Figure 4:**
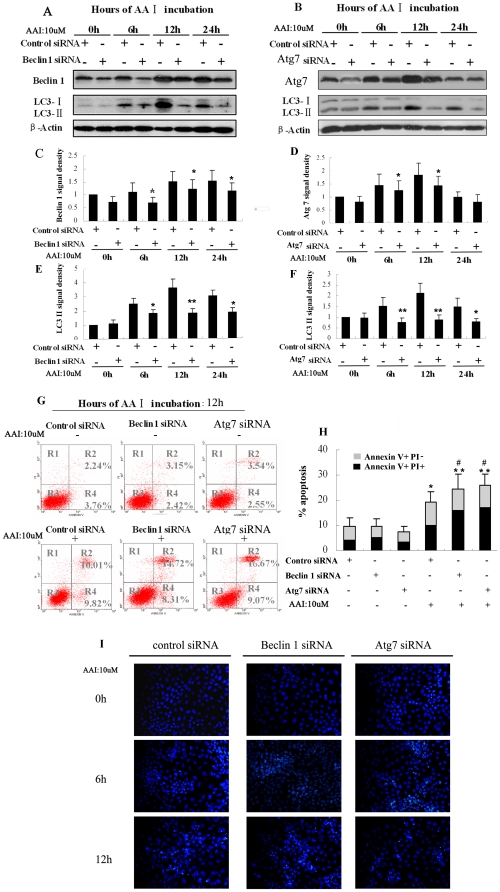
Increased apoptotic and necrotic cells after knockdown of Beclin 1 or Atg7 in NRK52E cells during AAI incubation. NRK52E cells were transiently transfected with negative control siRNA, Beclin 1 siRNA and Atg7 siRNA. The transfected cells were then treated with AAI (10 µM) for 0 to 24 hrs. **A**, Representative immunoblot images of Beclin 1 and LC3-II. **B**, Representative immunoblot images of Atg7 and LC3-II. **C**, Densitometric analysis of Beclin 1 in Fig. 4A. **D**, Densitometric analysis of Atg7 in Fig. 4B. **E**, Densitometric analysis of LC3II of Fig. 4A. **F**, Densitometric analysis of LC3II in Fig. 4B. **G**, Representative annexinV/PI flow cytometry analysis of negative control siRNA, Beclin 1 siRNA and Atg7 siRNA transfected cells with or without AAI treatment for 12 hrs. **H**, Quantitative analysis of AnnexinV+PI− and AnnexinV+PI+ NRK52E cells by flow cytometry. **I**, Typically morphological apoptotic cells images at different time (×600). **C**,**D**,**E**,**F** and **H**:Data was expressed as means±SEM of three independent experiments. *and** denote *p<0.05* and *p<0.001*, respectively, when compared to control siRNA without AAI treatment, respectively. ^#^ denote *p<0.05*, when compared to control siRNA with AAI treatment.

To further confirm the role of autophagy on cell fate, the effects of Beclin 1 and Atg7 knockdown on AAI-induced apoptosis in NRK52E cells were examined. NRK52E cells were transfected with siRNA of Beclin 1, siRNA of Atg7 or a negative control siRNA, respectively. The cells were then treated with AAI (10 µM) from 0 hr to 24 hrs. Compared with negative control siRNA interference, Beclin 1 siRNA or Atg7 siRNA interference remarkably abrogated the AAI-induced autophagy, which was obvious at 12 hrs (Fig. 4A–F). AAI-induced cell apoptosis and death were evaluated by Annexin V and PI assay as well as Hoechst33342 staining. As shown in figure 4G and 4H, there wasn't significant difference in cell apoptosis among negative control siRNA group, Beclin 1 siRNA group and Atg7 siRNA group under full culture condition without AAI treatment. The transfected cells were then subjected to 12 hrs of AAI treatment. Compared with the negative control siRNA group, knockdown of Beclin 1 or Atg7 increased cell apoptosis significantly after AAI treatment, especially for late apoptotic and necrotic cells (Fig. 4G and 4H). The morphology study of apoptotic cells by Hoechst33342 offered further evidence (Fig. 4I). In negative control siRNA group, few apoptotic cells were detected morphologically when cells were exposure on AAI for 6 hrs, while several clusters of apoptotic cells were observed in Atg7 siRNA and Beclin 1 siRNA group at the same time point (Fig. 4I). Compared with negative control siRNA group, more cells both in Beclin 1 siRNA group and Atg7 siRNA group developed to apoptotic death at 12 hrs, which exhibited as some adherent apoptotic cells exfoliated from coverslips and couldn't be fixed and detected by morphology. These results provided evidence that autophagy related gene siRNA interference could inhibit LC3-II formation and accelerate AAI induced cell death.

### AAI induced autophagy via activation ERK1/2 pathway

**Figure 5:**
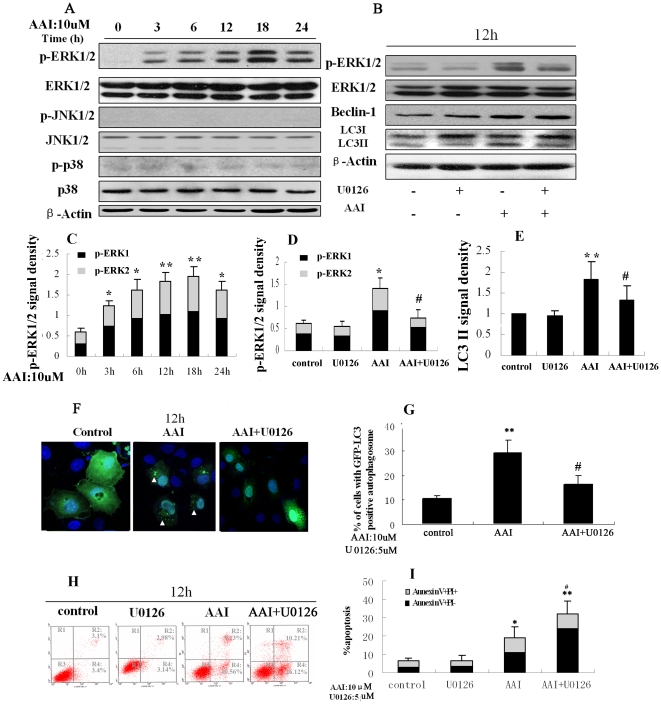
AAI induced autophagy via activation ERK1/2 pathway. NRK52E cells were treated with AAI (10 µM) for 0 to 24 hrs. **A**, Representative immunoblot images of p-ERK1/2, ERK1/2, JNK1/2, p-JNK1/2, p–p38 and p38 proteins. ERK1/2 pathway was activated at early stage, but not JNK and P38. U0126 inhibited activation of ERK1/2 pathway and accumulation of LC3II induced by AAI. NRK52E cells were incubated for 12 hrs in the absence (DMSO control), or presence of AAI (10 µM), U0126 (5 µM) or AAI plus U0126. **B**, Representative immunoblot images of p-ERK1/2, ERK1/2, Beclin 1 and LC3II proteins. **C**, Densitometric analysis of p-ERK1/2 in Fig. 5A. **D**, Densitometric analysis of p-ERK1/2 in Fig. 5B. **E**, Densitometric analysis of LC3II in Fig. 5B. **F**, Representative images (×800). Arrowheads indicate GFP-LC3 puncta (green). Nuclei (blue) were stained by Hoechst33342. **G**, Graphs represent quantitation analysis of the percentage of cells with GFP-LC3 positive autophagosomes. U0126 increased cell apoptosis induced by AAI. **H**, Representative annexinV/PI flow cytometry analysis of cells from DMSO control, AAI, U0126 and AAI plus U0126 groups. **I**, Quantitative analysis of AnnexinV+PI− and AnnexinV+PI+ NRK52E cells by flow cytometry. **C**,**D**,**E**,**G** and **I:** Data was expressed as means±SEM of three independent experiments. *and** denote *p<0.05* and *p<0.001*, respectively, when compared to control condition. ^#^denote *p<0.05*,compared to AAI group.

Under the same incubation condition of AAI, phosphorylation level of mitogen-activated protein kinases (MAPKs), including ERK1/2, p38, and JNK proteins were detected by western blot analysis. AAI induced a significantly activation of ERK1 and ERK2 in a time-dependent way which began at 3 hrs and peaked at 18 hrs (Fig. 5A and 5C). However, 10 µM AAI exposure had little effect on the activation JNK and p38 in NRK52E cells (Fig. 5A). To further investigate the effect of ERK1/2 activation on autophagy, ERK1/2 was blocked with U0126 (5 µM), an ERK1/2 inhibitor. U0126 itself didn't alter accumulation of LC3-II protein, but attenuated AAI-induced LC3-II protein accumulation partially (Fig. 5B and 5E). The immunofluorescence offered another evidence, in which pre-treatment with U0126 resulted in a decreased autophagosome formation (Fig. 5F and 5G). These results demonstrated that ERK1/2 signaling pathway involved in AAI induced-autophagy. At last, the effect of U0126 on cell apoptosis induced by AAI was detected. U0126 itself didn't affect cell apoptosis, but significantly increased AAI-induced apoptosis in NRK52E cells (Fig. 5H and 5I).

## Discussion

Despite some autophagy research were done in other renal diseases [5], [21], [22], information about autophagy in AAs-induced renal injury was absent. The current study has characterized autophagy during AAI-induced renal tubular epithelium cell injury. We demonstrated for the first time that low dose of AAI (10 µM) induced autophagy in NRK52E cells as early as 3 hrs, and maintained at high level for 12 hrs, which was indicated by punctuate GFP-LC3 localization and LC3-II formation. It was reported that an increase in autophagosomes, the morphologic hallmark of autophagy, in cultured renal tubular cells under hypoxic condition as well as cisplatin induced injury. Our results accorded with these studies concerning renal tubular injury and autophagy. Besides, we confirmed that AAI didn't block autophagic flux, but induced autophagic activity by observing the accumulation of LC3 II with or without lysosomal protease inhibitors. We also found AAI induced scarce apoptotic cells until 12 hr. These results clearly confirmed that AAI-induced autophagy occurred prior to apoptosis in proximal tubules.

Whether autophagy enhances or diminishes the apoptotic response is a debated issue. Indeed, investigating the interplay between autophagy and apoptosis is beneficial if the autophagy pathway is to be targeted effectively in the treatment of disease. Under the AAs induced-injury, renal tubular epithelial apoptosis is a mainly pathophysiological mechanism, which leads to tubular epithelial cell deletion and tubular atrophy [11], [23]. The next question we addressed was whether this induction of autophagy observed in the AAI induced-cell injury was specific to affect the fate of cells. We demonstrated that inhibition of autophagy with Wortmannin or 3-MA, two effective autophagy inhibitors, significantly increased AAI induced apoptosis rate, whereas Wortmannin and 3-MA themselves didn't affect cell apoptosis. Beclin 1 and Atg7 are autophagy-related genes which are two type key genes for formation of autophagy [24], [25]. To further confirm the effect of autophagy, small-interfering RNA knockdown of Beclin 1 or Atg7 was used to block autophagy in cultured NRK52E cells, respectively. It was shown that down-regulation of Beclin 1 or Atg7 decreased AAI-induced LC3-II formation. Compared with negative control siRNA group, both Beclin 1 siRNA and Atg7 siRNA enhanced apoptosis during AAI incubation as indicated by apoptotic cells detected as early as 6 hrs and more apoptotic cell death detected at 12 hrs after AAI treatment. Taken together, these data support that autophagy protect renal tubular cells from apoptosis under AAI toxicity and play an essential prosurvival role in AA-induced renal tubular cell injury.

Wang, et al. reported the AMPK-MEK/ERK-TSC-mTOR pathway regulation of Beclin 1 represented different thresholds responsible for a protective or destructive autophagy [26]. Sivaprasad *et al.* confirmed that inhibition of ERK1/2 in MCF-7 cells resulted in decreased autophagy in response to TNF, accompanied with increasing sensitivity of cell death [27]. In consistent with these results, we demonstrated in this study that AAI induced autophagy in NRK52E cells via the ERK1/2 pathway, which indicated by an upregulation of pERK1/2 protein expression at early stage and an attenuated accumulation of LC3-II by inhibitor of ERK1/2. Although JNK and p38 played a critical role in autophagy induced by cellular starvation and cell swelling [28], [29], our study couldn't observe the activation of JNK and p38 during AAI induced-cell injury.

In summary, our study demonstrated for the first time that autophagy occurred earlier than apoptosis during AAI-induced tubular epithelial cell injury. Autophagy induced by AAI via ERK1/2 pathway might attenuate apoptosis, which may provide a protective mechanism for cell survival under AAI-induced pathological condition. More studies have to be performed to understand how autophagic and apoptotic responses by AAI orchestrate to contribute to the AAN.
